# Ultrasound imaging of carotid web with atherosclerosis plaque: a case report

**DOI:** 10.1186/s13256-020-02446-1

**Published:** 2020-09-08

**Authors:** Bin Ning, Dong Zhang, Binbin Sui, Wen He

**Affiliations:** 1 Department of Ultrasound, Beijing Tiantan Hospital, Capital Medical University, No. 119, 4th South Ring West Road, Fengtai District, Beijing, 100070 China; 2 Department of Neurosurgery, Beijing Tiantan Hospital, Capital Medical University, No. 119, 4th South Ring West Road, Fengtai District, Beijing, 100070 China

**Keywords:** Carotid web, Atherosclerosis plaque, Stroke

## Abstract

**Background:**

To the best of our knowledge, no previous studies on carotid webs with atherosclerosis plaque have been conducted. Thus, both radiologists and clinicians have insufficient knowledge of this disease, which could lead to misdiagnosis and missed diagnosis. An accurate diagnosis is beneficial to clinical management and prevention of stroke. Here, we present a case of a carotid web with an atherosclerotic plaque, which was confirmed by histopathology and was treated at the Department of Neurosurgery, Beijing Tiantan Hospital.

**Case presentation:**

We report a rare case of a carotid web with an atherosclerotic plaque in a 61-year-old Han man. He presented to our hospital with history of intermittent dizziness and slurred speech for 1.5 years and numbness of both upper limbs for 4 months. A computed tomography angiography examination indicated severe stenosis at the beginning of the left internal carotid artery with plaque surface ulceration. Doppler ultrasound examination showed a carotid web with a thin isoechoic plaque and a membrane-like structure protruding into the lumen from the lateral posterior wall at the beginning of the left internal carotid artery. The thin isoechoic plaque could be seen at the base of the membrane-like structure. Carotid endarterectomy was performed to alleviate symptoms. A carotid web with atherosclerosis was diagnosed intraoperatively, and postoperative pathology confirmed extensive intima fibroid hyperplasia accompanied with myxoid degeneration. The base of the carotid web was attached to the thin atherosclerosis plaque, and between the web and the plaque, a cavity was observed. In this case report, we aim to discuss the diagnosis of carotid web with atherosclerosis, its physiopathology and management, and the possible reasons for missed diagnosis or misdiagnosis.

**Conclusion:**

Carotid webs with atherosclerosis have no known etiological factors and are rarely reported. Thus, carotid webs could be easily confused with ulcerations on the surface of the atherosclerosis plaque. The diagnosis could be difficult and effective management remains indeterminate. Moreover, prompt recognition of this disease is key to correct treatment and management. Hence, this case report and the relevant data in the literature could contribute to the improvement of the diagnosis and treatment of this disease.

## Background

Carotid webs with atherosclerotic plaques are one cause of stroke; however, misdiagnosis and missed diagnosis of this disease are frequent. Its diagnosis is a challenge for radiologists and sonographers because it is rare and relevant reports are scarce, which in turn affects the treatment and management of the disease. Although previous reports of carotid web exist [1–18], carotid webs with atherosclerotic plaque formation have not been mentioned in previous studies. Thus, establishing the image characteristics of this disease is vital to improve treatment and outcomes. In this report, we aim to present a case of and provide further information on a carotid web with an atherosclerotic plaque; pathological findings were confirmed after carotid endarterectomy (CEA).

## Case presentation

A 61-year-old Han man presented to our hospital with intermittent dizziness and slurred speech for 1.5 years and numbness of both upper limbs for 4 months. After resting, he showed improvement and had intermittent attacks without any treatment. A computed tomography angiography (CTA) examination indicated stenosis at the beginning of the left internal carotid artery. Because of recurrent symptoms, he sought admission to our hospital for further treatment. Moreover, he had diabetes for 17 years and was treated with an orally administered antidiabetic medication. He also had hypertension for 10 months (blood pressure up to 220/120 mmHg) and was treated with an orally administered antihypertensive drug. He had 20-pack years of smoking history.

### Doppler ultrasound (DUS) and CTA examination

**Fig. 1:**
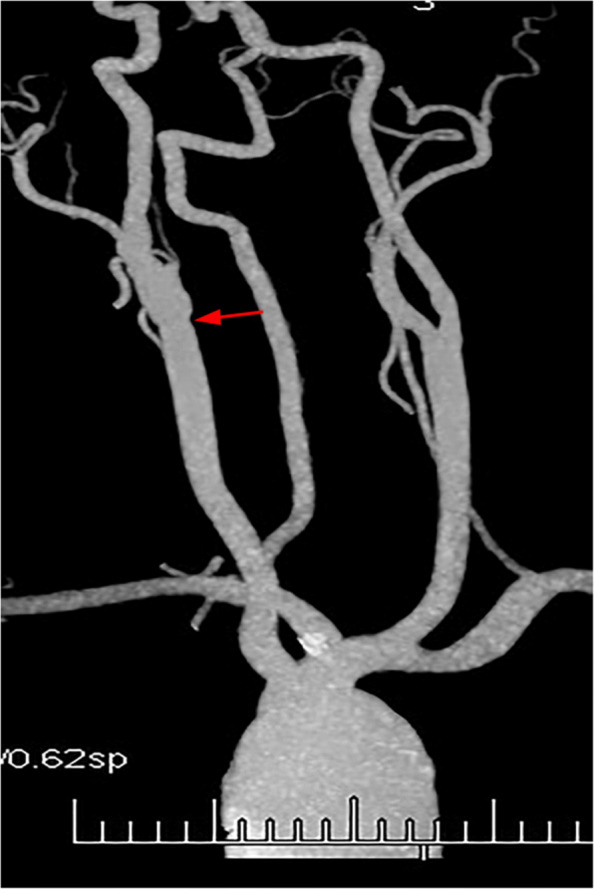
Computed tomography angiography indicated a small niche shadow in the left internal carotid artery on sagittal view (*red arrow*)

**Fig. 2:**
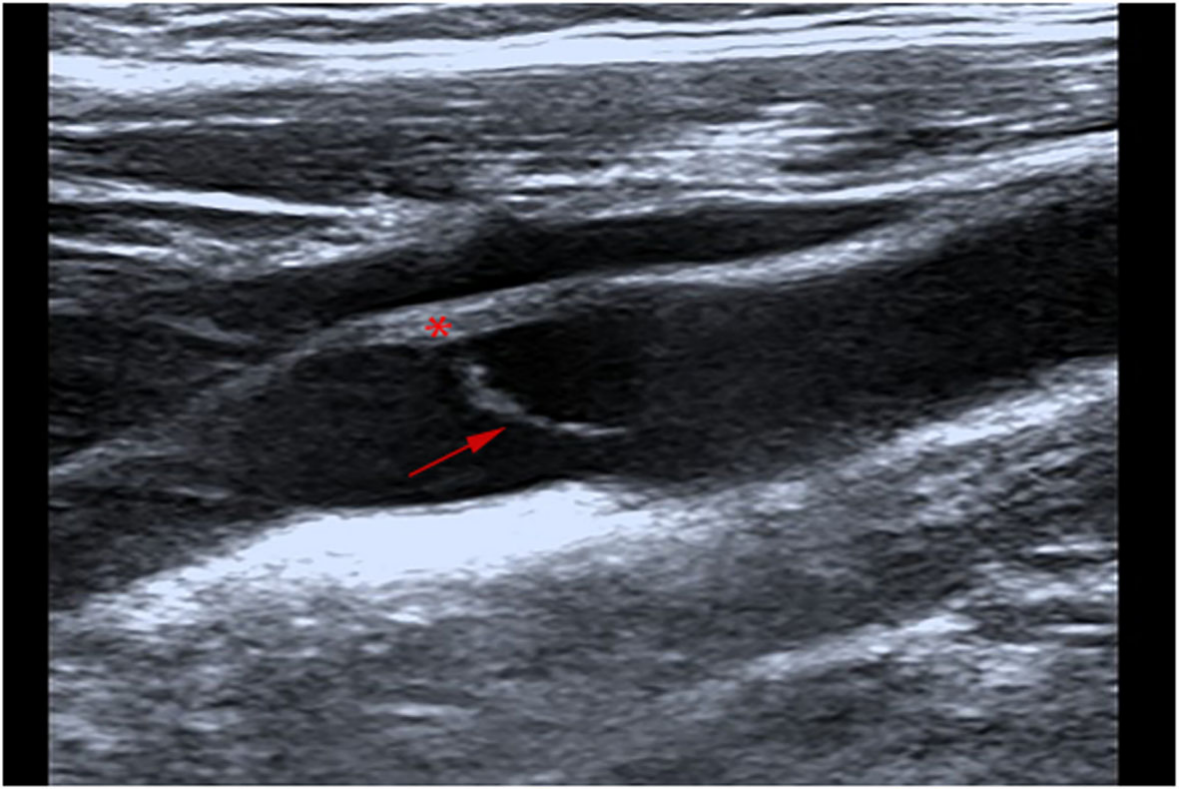
Doppler ultrasound showing membrane-like structure protruded into the lumen (*arrow*). The isoechoic plaque (*star*) on the artery wall was attached to the basilar part of the membrane-like structure

**Fig. 3:**
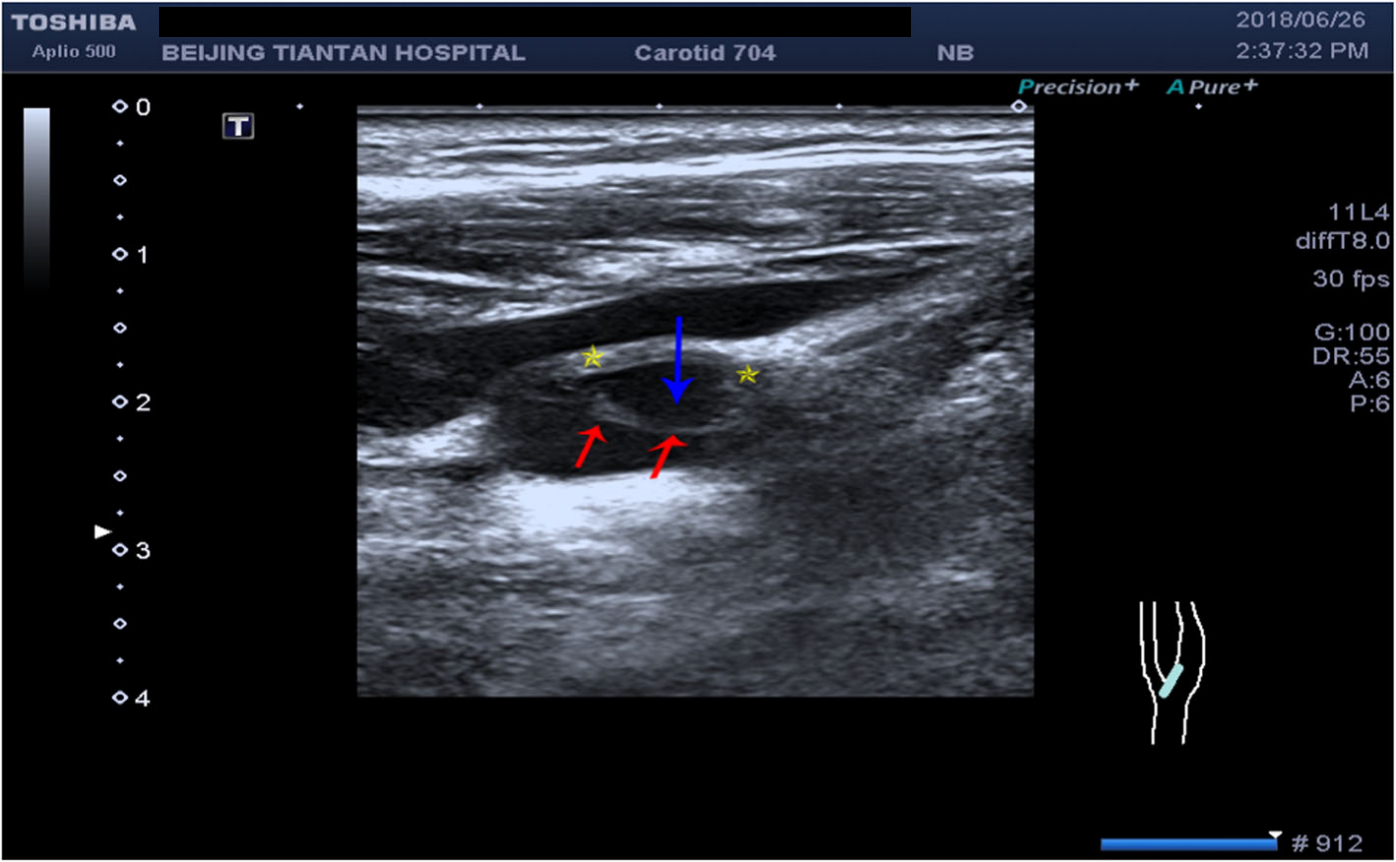
Doppler ultrasound showing carotid web (*red arrow*), plaque (*yellow star*), and huge cavity (*blue arrow*)

**Fig. 4:**
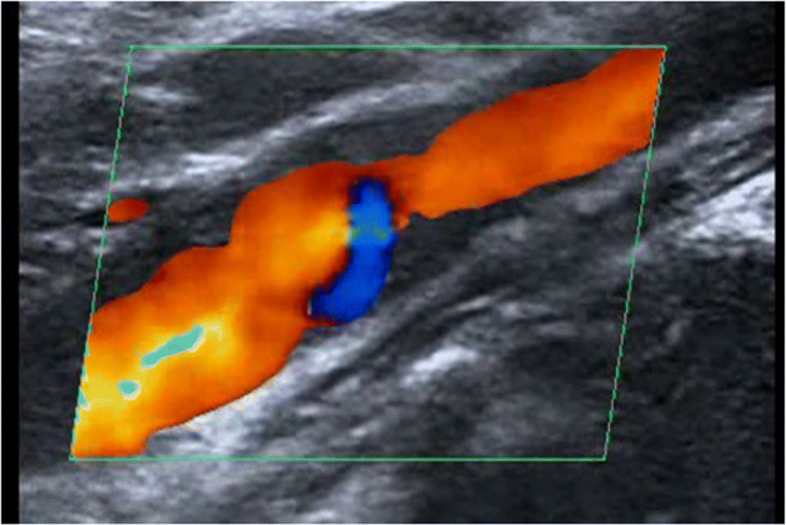
Color Doppler flow image showing whirlpool at the level of the carotid web on the longitudinal section

**Fig. 5:**
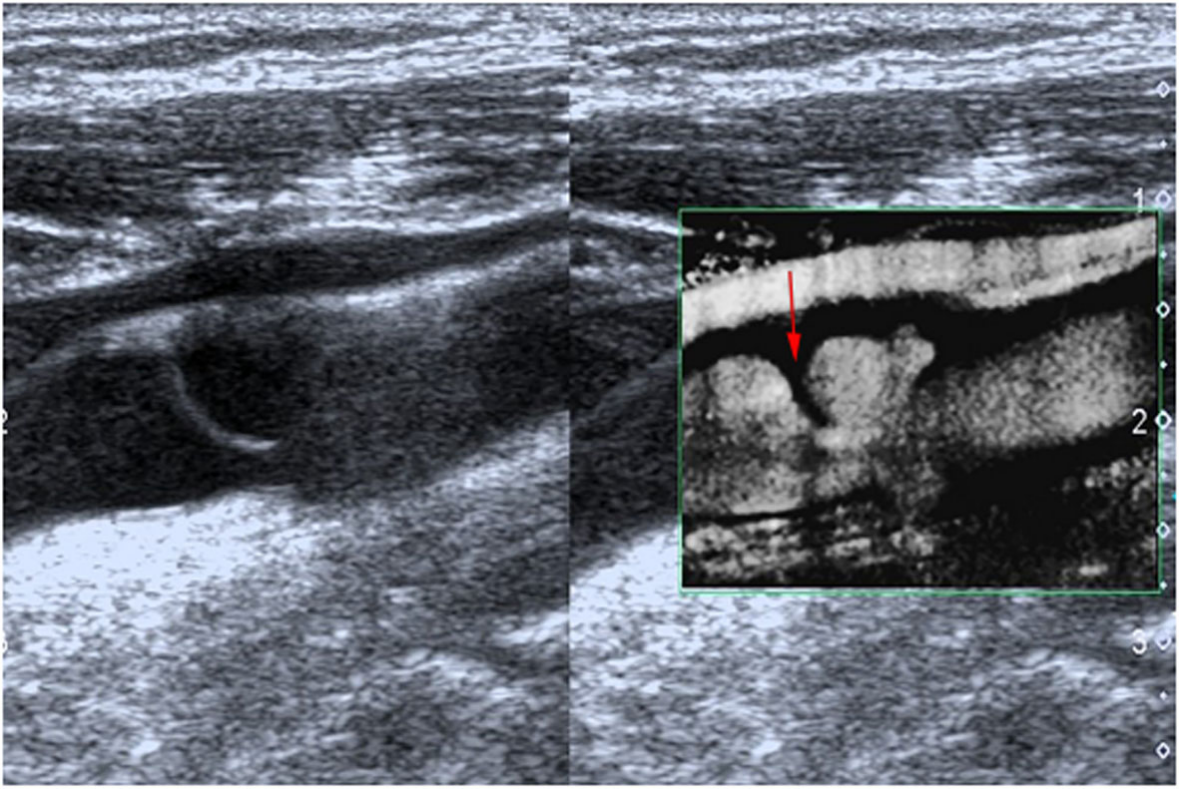
Superb micro vascular imaging showing the membrane-like filling defect of the carotid web (*arrow*) on longitudinal view

**Fig. 6:**
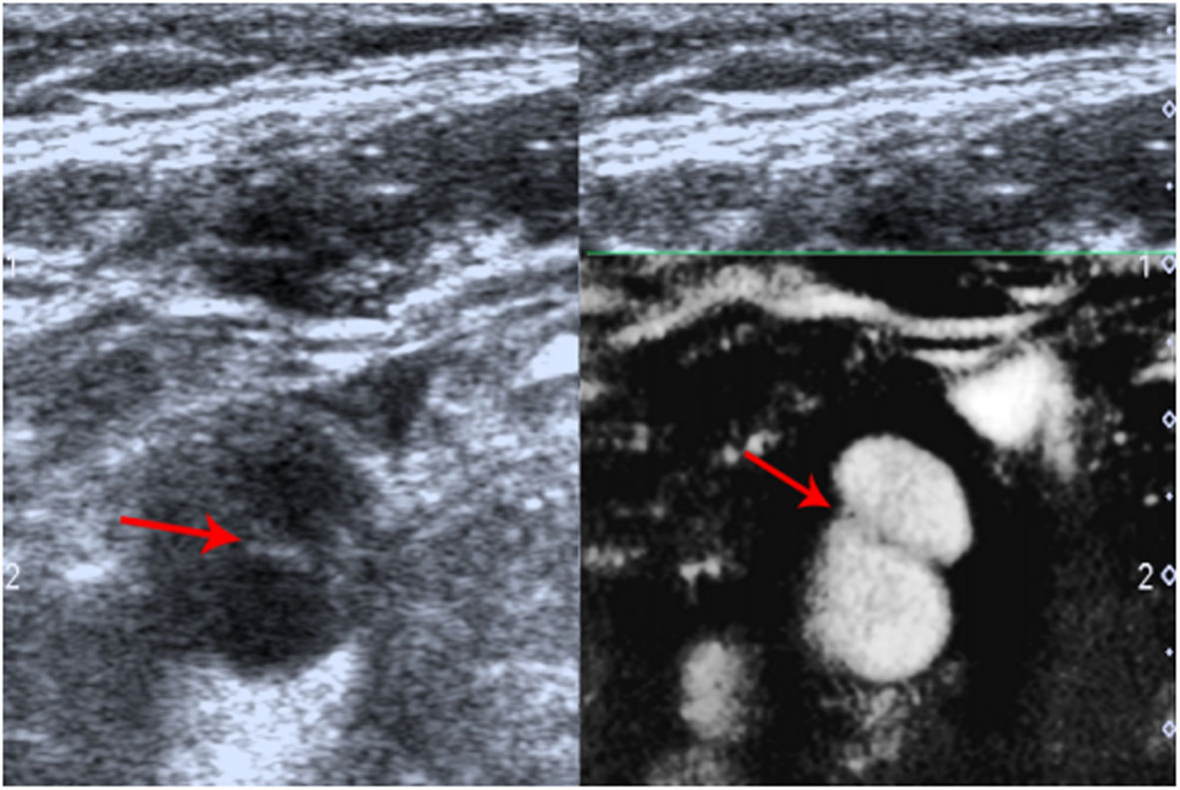
Superb micro vascular imaging showing the carotid web on transverse view (*arrow*)

Routine examination with CTA and DUS before surgery was performed. CTA indicated a small niche shadow in the left internal carotid artery on sagittal view (Fig. 1), and no significant stenosis was found. The diagnosis based on CTA was atherosclerosis plaque surface ulceration. In the preoperative ultrasound examination, a membrane-like structure protruding into the lumen from the lateral posterior wall at the beginning of the left internal carotid artery on gray scale was noted, and an isoechoic plaque could be seen at the basilar part of the membrane-like structure (Fig. 2). Moreover, the membrane-like structure projected into the lumen in a certain curve and was not floating in the blood. We adjusted the scanning angle for a better view of the whole length of the membrane-like structure; we observed a huge hollow space between the membrane-like structure and the plaque, which was similar to a large ulcer; however, the plaque surface was smooth and flat (Fig. 3). In addition, color Doppler flow image (CDFI) showed a whirlpool at the level of the carotid web (Fig. 4), and superb micro vascular imaging (SMI) demonstrated a membrane-like filling defect with a small, broad base in the longitudinal (Fig. 5) and transverse (Fig. 6) views. A diagnosis of a carotid web with an atherosclerotic plaque was made based on the aforementioned image characteristics. Furthermore, spectral Doppler imaging was performed on an ultrasonic imaging system (TOSHIBA Aplio™ 500, Japan) equipped with a linear array transducer (11 L-4 probe) at the carotid preset (frequency = 8 MHz, wall filter = 5).

### Treatment and histopathology

**Fig. 7:**
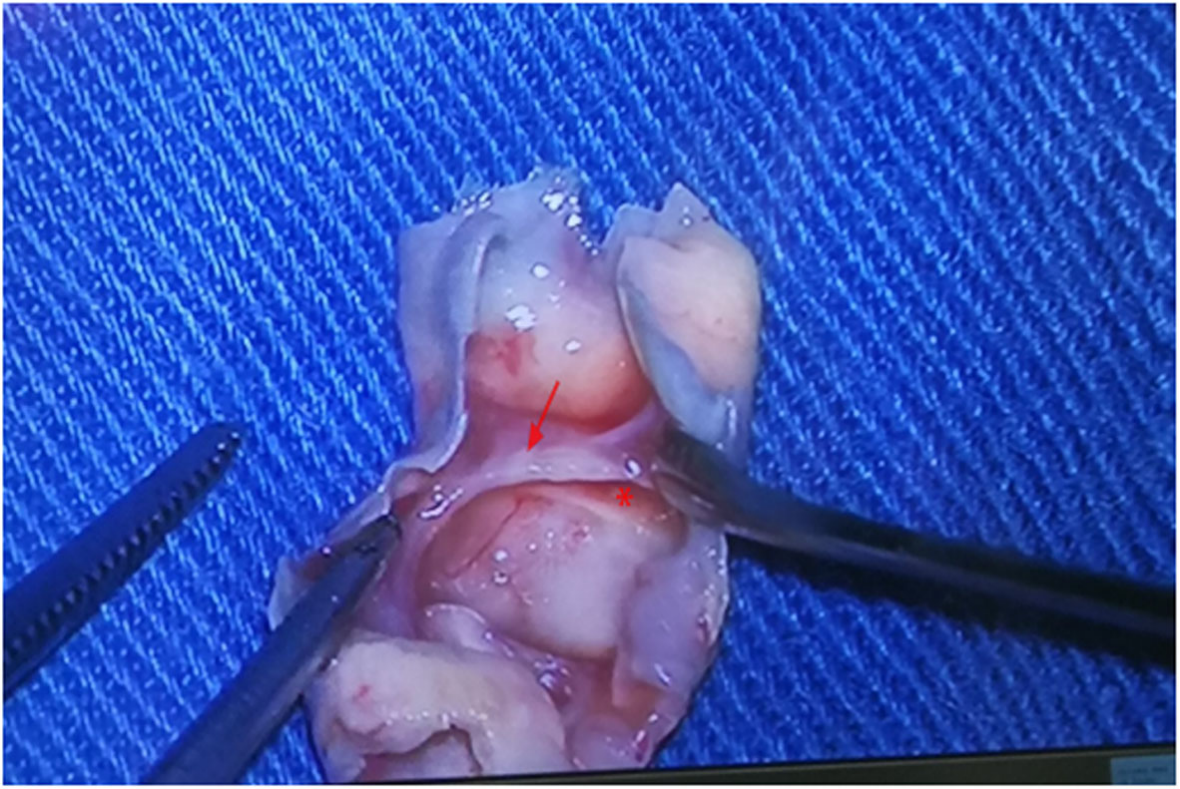
Macroscopic view of surgical specimen. The carotid web (*red arrow*), atherosclerosis plaque (red star), and any part of the tissue were smooth. No ulcer on the surface of plaque or carotid web was noted

**Fig. 8:**
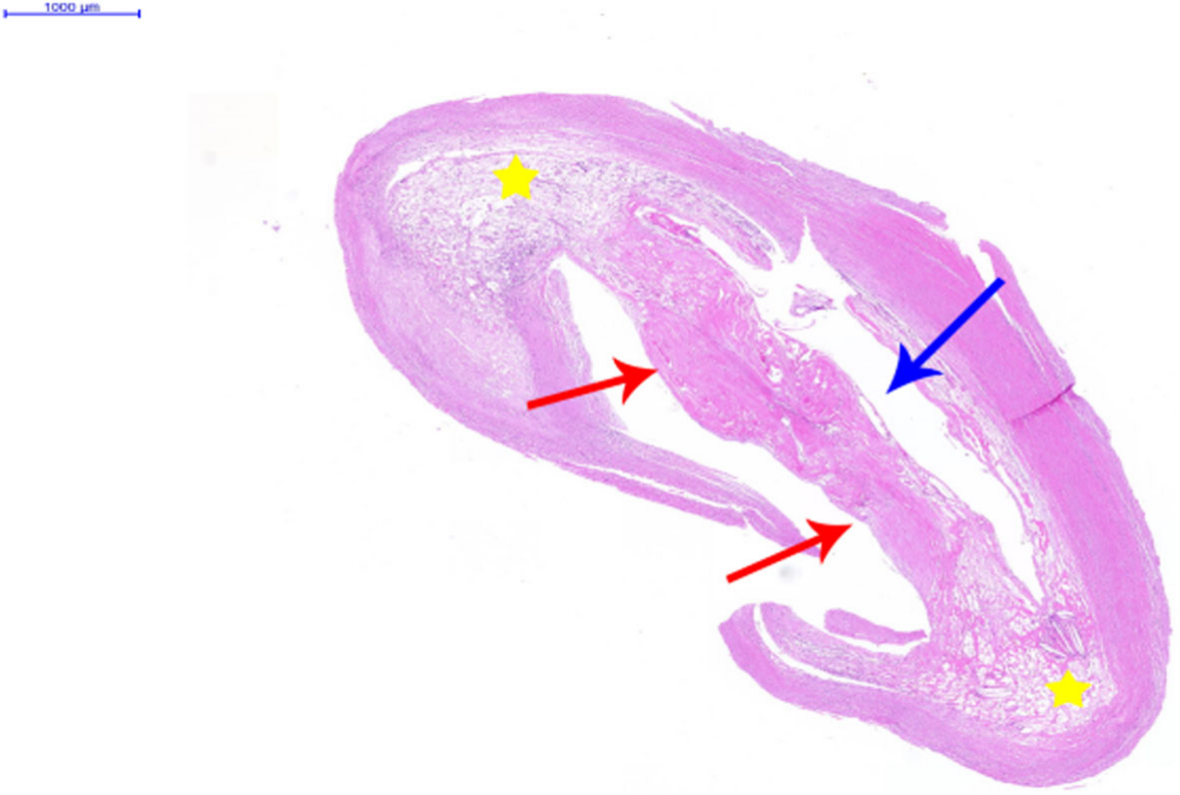
Hematoxylin-eosin staining confirmed that carotid web (*red arrow*) is extensive intima fibroid hyperplasia with plaque (*yellow star*) and cavity (*blue arrowhead*) between the carotid web and plaque

**Fig. 9:**
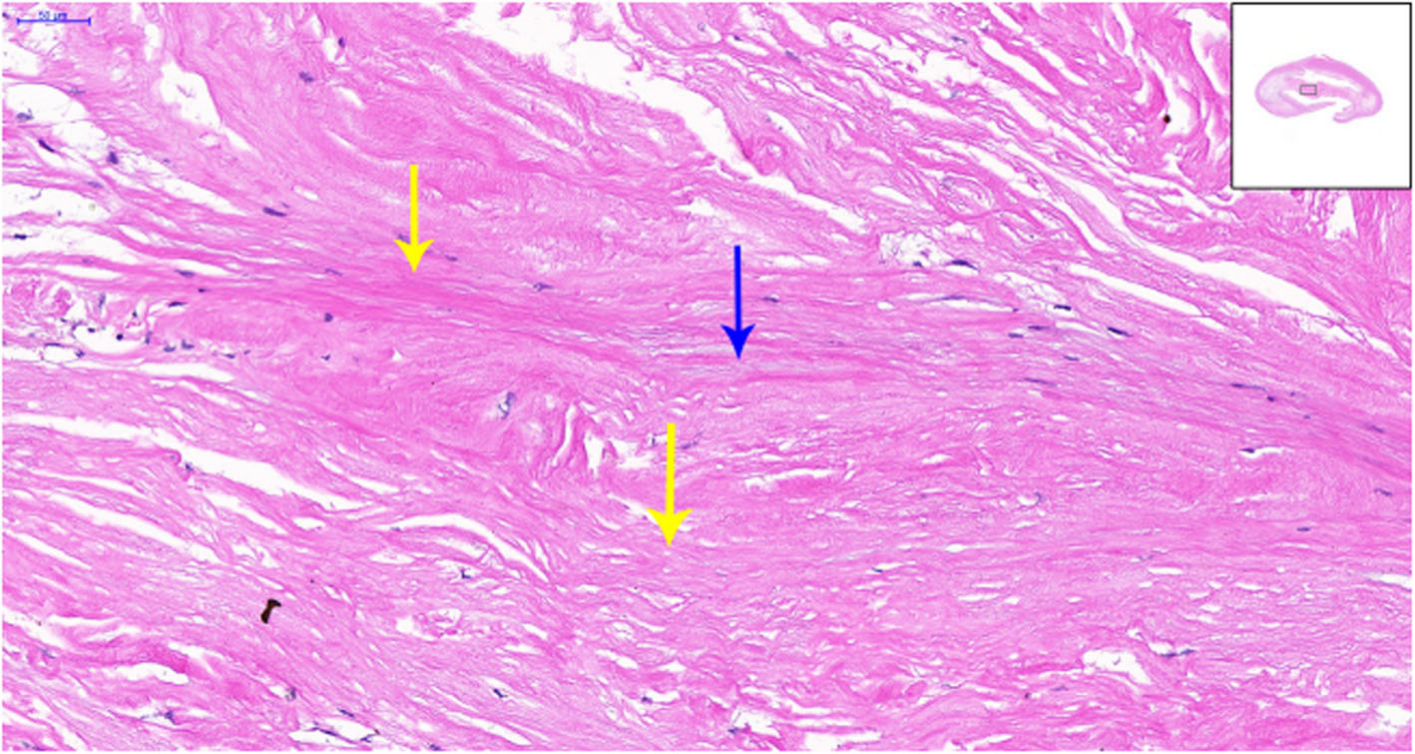
The carotid web was extensive intima fibroid hyperplasia (*yellow arrow*) with myxoid degeneration (*blue arrow*) (high magnification)

**Fig. 10:**
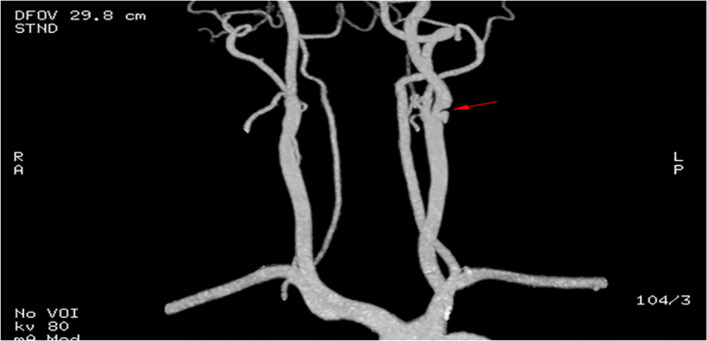
Multiplanar reconstruction of computed tomography angiography (after carotid endarterectomy) showed membrane-like filling defect (*red arrow*) on sagittal view

**Fig. 11:**
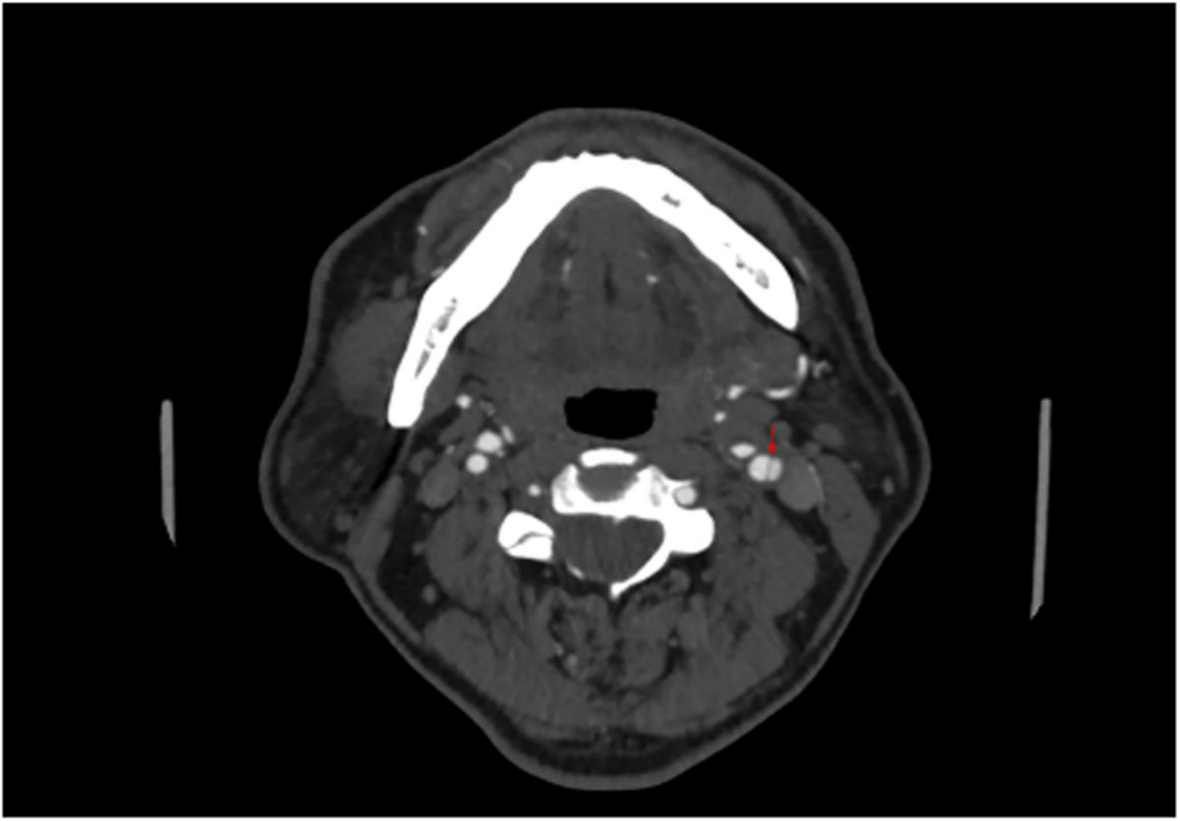
Multiplanar reconstruction of computed tomography angiography (after carotid endarterectomy) showed membrane-like filling defect (*red arrow*) on axial view, which could be easily confused with artery dissection or ulcer

To relieve the symptoms of our patient, CEA was performed. The diagnosis of a carotid web with an atherosclerotic plaque by DUS was confirmed by the postoperative specimen (Fig. 7); both the carotid web and the plaque surface were smooth without evidence of ulceration, which was consistent with the findings of DUS. The lesion tissue after CEA was fixed in formalin, embedded in paraffin, and sectioned in the axial plane. Sections were stained for hematoxylin and eosin, and the postoperative gross specimen and histopathology showed that the basilar part of the carotid web contained an atherosclerosis plaque (Figs. 7 and 8). The carotid web consisted of extensive intima fibroid hyperplasia with myxoid degeneration (Fig. 9); moreover, no ulceration was found in any of the sections. Subsequently, we performed CTA multiplanar reconstruction, and the membrane-like filling defect was best shown in both the sagittal and axial views (Figs. 10 and 11); however, the atherosclerosis plaque attached to the carotid web could not be observed clearly. Our patient’s neurologic status continued to improve postoperatively.

## 3. Discussion and conclusions

To understand carotid webs with plaque formation, we should first fully understand a carotid web. The term “web” was first used to describe this disease by Momose and New in 1973 at the Massachusetts General Hospital [7]. They analyzed 7000 carotid angiograms for 8 years and found 4 cases with web-like tissue in the lumen in the cervical portion of the internal carotid artery [7]. Subsequently, relevant studies on carotid webs were reported [1–5], which suggested that carotid webs are thin membrane structures protruding into the lumen; they are more common in the posterior or lateral posterior wall of the internal carotid bulb, and are approximately eight times more common in patients with stroke than in controls. Moreover, carotid webs are strongly associated with ischemic stroke [3, 8], and they are one of the causes of cryptogenic stroke in young patients [1, 4, 10, 15]. However, they are often missed [11] and could be an underappreciated risk factor for stroke [4, 9]. In addition, carotid webs may contribute to recurrent ischemic stroke [6, 9, 10], despite antithrombotic use and antiplatelet therapy. It is worth emphasizing that the image characteristics of a carotid web with atherosclerotic plaque formation could be easily confused with plaque surface ulceration, especially in geriatric patients. Both radiologists and sonographers may not consider the possibility of this disease and thus make the wrong diagnosis, that is, plaque surface ulcer formation. However, the two diseases are treated differently. Carotid webs with or without atherosclerotic plaques should be treated with antiplatelet therapy, and the most effective and radical treatment is surgical intervention, whereas the treatment of atherosclerosis plaque surface ulceration may include plaque stabilization therapy. Thus, accurate diagnosis is key to preventing strokes and treating the disease. We consider that ultrasound has absolute diagnostic advantages, although reports on the disease diagnosed by ultrasound examination are rare. However, Kliewer and Carroll [13] and Perren F *et al*. [14] described carotid webs using DUS in 1991 and 2004, respectively, and another case of carotid web by DUS was reported in 2015 [11]. While almost all diagnoses and reports on this disease were based on CTA or digital subtraction angiography, a study in 2017 using DUS reported on a carotid web with a short segment filling defect [15]. Thus, DUS could make an accurate diagnosis and may have the ability to diagnose carotid web. However, an accurate diagnosis depends on the full understanding and careful observation of the image characteristics. In our case, we found that the carotid web with a plaque had similar image characteristics to plaque surface ulcer formation due to the cavity structure; however, substantial differences between the two diseases were noted. The top of ulcer cavities has no membranous structure, while the plaque attached to the base of the carotid web did have a membranous structure on the cavity surface.

Moreover, DUS could show the carotid web and the plaque clearly, that is, the structural and positional relationships between the carotid web and plaque could be clearly displayed on ultrasound. However, slight adjustment of the probe direction is needed to trace the direction and angle change of the membrane structure, and careful observation on multiple angles is necessary. Moreover, high suspicion of the disease may be helpful for the diagnosis. In our case, a minor niche shadow on the lateral wall at the beginning of the internal carotid artery was identified with CTA (Fig. 1) and the carotid web was not found. Therefore, the absence of any suspicion and insufficient recognition of this disease may be the reasons for the misdiagnosis with CTA. After CEA, we observed the fresh tissue and histopathology features of the disease and summarized the image characteristics based on DUS and made a CTA image reconstruction. Interestingly, the manifestations of the carotid web with plaque based on DUS and CTA image reconstruction were similar (Figs. 5, 6, 10, and 11). The filling defect was membrane-like or shelf-like and a cavity was found between the web and the plaque, which could be easily confused with plaque surface ulceration. However, the disease differs from plaque surface ulceration as the surface of the ulcer is eroded and damaged and does not have intact smooth endothelium, whereas the surface of the carotid web with an atherosclerotic plaque is smooth and intact.

Clinicians, including radiologists and sonographers, should be aware that carotid webs with atherosclerotic plaques could occur not only in young patients with stroke but also in elderly patients with stroke. Because the pathogenesis of carotid webs alone versus carotid webs with atherosclerotic plaques are similar, accurately distinguishing the two diseases presents a diagnostic and therapeutic challenge. Thus, careful examination of the diseases is vital to decrease missed diagnosis and misdiagnosis. A previous study reported that early and correct diagnosis of carotid webs is crucial not only to establish the probable cause of stroke but also to prevent the risk of recurrence [2]; thus, adequate understanding of this disease for early diagnosis is critical for the prevention of stroke and recurrence, and for the clinical management of the disease.

However, currently, carotid webs are challenging because of their small and thin characteristics. The low detection rate may be due to a lack of experience, insufficient scanning skills, and limited understanding of the image characteristics. To reduce the rate of missed diagnosis and misdiagnosis, both radiologists and sonographers should increase their familiarization of the image features of the disease. Moreover, acoustic beam direction should be traced along the direction of the carotid web, the whole length evaluated, and the basilar part of the carotid web carefully observed to detect whether the base is attached to the plaque or has thrombus formation. Lastly, high suspicion of the disease could aid in the diagnosis.

In conclusion, DUS could show the morphology of the carotid web with or without plaques; however, improvement in scanning skills is vital. CTA was considered the gold standard for the diagnosis of this disease; however, without adequate knowledge, it could lead to misdiagnosis and missed diagnosis. Early diagnosis of the disease could guide the clinical management of the disease, thereby reducing the incidence and recurrence of stroke.

## Data Availability

The datasets used and/or analyzed during the current study are available from the corresponding author on reasonable request.

## Abbreviations

CEA: Carotid endarterectomy
CTA: Computed tomography angiography
DUS: Doppler ultrasound
